# Gold nanorods with conjugated polymer ligands: sintering-free conductive inks for printed electronics†

**DOI:** 10.1039/c6sc00142d

**Published:** 2016-03-15

**Authors:** B. Reiser, L. González-García, I. Kanelidis, J. H. M. Maurer, T. Kraus

**Affiliations:** a INM – Leibniz Institute for New Materials Campus D2 2 66123 Saarbrücken Germany lola.gonzalez-garcia@leibniz-inm.de tobias.kraus@leibniz-inm.de

## Abstract

Metal-based nanoparticle inks for printed electronics usually require sintering to improve the poor electron transport at particle–particle interfaces. The ligands required for colloidal stability act as insulating barriers and must be removed in a post-deposition sintering step. This complicates the fabrication process and makes it incompatible with many flexible substrates. Here, we bind a conjugated, electrically conductive polymer on gold nanorods (AuNRs) as a ligand. The polymer, poly[2-(3-thienyl)-ethyloxy-4-butylsulfonate] (PTEBS), provides colloidal stability and good electron transport properties to stable, sintering-free inks. We confirm that the polymer binds strongly through a multidentate binding motif and provides superior colloidal stability in polar solvents over months by IR and Raman spectrometry and zeta potential measurements. We demonstrate that the developed ligand exchange protocol is directly applicable to other polythiophenes such as poly(3,4-ethylenedioxythiophene):polystyrene sulfonate (PEDOT:PSS). Films of AuNRs coated with above polymers reached conductivities directly after deposition comparable to conventional metal inks after ligand removal and retained their conductivity for at least one year when stored under ambient conditions.

## Abstract 2

A straightforward route to hybrid nanostructures of metal cores with conductive polymers and their application as sintering-free inks is described.

## Graphical abstract

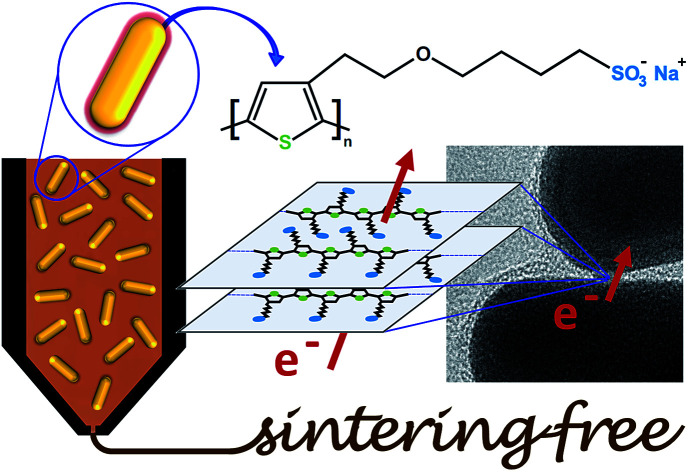

## Introduction

The accelerating market of printed electronics requires inks that are suitable for large-area, high-throughput and low-cost production of lightweight and flexible conductive materials. Relevant market drivers are touchscreen panels, memory components, organic photovoltaic, radio frequency identification (RFID) tags and optoelectronic devices.^1^ Preferable deposition methods are solution-based processes such as inkjet printing using inks containing metal or conductive metal oxide colloids.^2–4^

Successful printing requires suitable inks. The performance of nanoparticle-based inks depends on their colloidal stability under the conditions that occur during printing.^1,5^ Conventionally, bulky organic molecules are used as ligands to ensure colloidal stability; they provide steric stabilization to the nanoparticles. After deposition, these ligands impede the contact between the particles and limit electrical conduction. Organic molecules represent insulating barriers; post-deposition treatments to remove them after drying are required. Thermal sintering at high temperatures and with long residence times is hard to reconcile with polymer substrates and roll-to-roll printing processes.^6^ Alternative post-treatment methods with reduced time and thermal budgets include plasma, laser, infrared (IR), microwave and intense pulsed light treatments.^1,7–10^ Some of them can remove organic ligands and improve electrical transport in less than a minute, but the resulting volume shrinkage can rupture the material.

Sintering-free inks avoid these challenges altogether. Grouchko *et al.* developed a self-sintering metal nanoparticle ink with a non-volatile destabilizing agent.^11^ Upon solvent evaporation, the concentration of this destabilizing agent increases and detaches the ligand from the particles. Detachment leads to metal–metal contacts, but the ink with its rapidly decreasing colloidal stability is hard to handle.

Here, we introduce a sintering-free nanoparticle ink in which conjugated polymer ligands lend the particles

• chemical stability due to strong multidentate binding to the metal surface,

• colloidal stability and compatibility in different relevant solvents, and

• good electron transport properties in dry state.

Kanehara and coworkers synthesized tailored phthalocyanine ligands and demonstrated that aromatic systems can provide mobile electrons in the ligand shells.^2,12^ We adapted this idea with polymer-coated particles and created hybrid particles with increased colloidal stability using commercially available conjugated polymers such as poly[2-(3-thienyl)-ethyloxy-4-butylsulfonate] (PTEBS) with an average molecular weight of 40–70 kDa. To ensure that the π-electrons couple to the metal surface, polythiophene derivatives were used; they bring the π-system in close proximity to the gold because they contain a sulfur heteroatom in the aromatic ring. Polymer chains with more than 100 repetition units, molecular weights of more than 20 kDa, and highly polar side chains can provide colloidal stability in polar solvents.

We demonstrate the effectiveness of polythiophene ligands in nanoparticle inks based on gold nanorods (AuNRs). AuNRs are anisotropic nanoparticles that show lower percolation thresholds than spherical particles and thus provide large conductivities at low volume fractions.^13^ The rods can be synthesized using a well-established protocol that yields narrow size distributions and negligible shape impurities.^14^ After synthesis, AuNRs are capped by a cetyltrimethylammonium bromide (CTAB) double layer (AuNR@CTAB).^15^ The ligand plays a crucial role in the anisotropic particle growth^14,16,17^ but leads to poor colloidal stability unless the AuNRs are kept in excess CTAB.^16^

Most existing strategies for the stabilization of AuNRs are based on large, non-conductive polymers that provide stability even if the CTAB has not been exchanged completely.^17–20^ The poor colloidal stability of AuNR@CTAB system renders ligand exchange with small molecules challenging.^16,18^ The few successful exchange protocols that exist for small ligands^17,21^ require unusual and non-conductive ligands or multi-step ligand exchange protocols. The rods' anisotropy presents an additional challenge: surface properties of the different crystal planes presented on the rod are not equivalent. It is possible to specifically exchange ligands only at the tips of the rods.^22–24^ For nanoparticle inks, ligand exchange protocols have to be chosen such that a homogeneous ligand shell forms unless anisotropic particle interactions during ink processing are desirable.

Here we describe a facile and straightforward ligand exchange procedure to modify AuNR@CTAB with PTEBS. We prove the complete exchange of the ligand and discuss the binding site and arrangement of the polymer chains on the surface of the AuNRs. The resulting colloidal dispersion was stable in water and in a mixture of polar solvents over months. We formulated inks and used them to deposit conductive patterns that immediately reached conductivities in the range of annealed metal inks. The protocol is also readily applicable to other polythiophenes, and we demonstrate its compatibility with poly(3,4-ethylenedioxythiophene):polystyrene sulfonate (PEDOT:PSS), a polymer mixture commonly used in organic electronics.

## Results and discussion

### Nanorod synthesis and ligand exchange

**Fig. 1:**
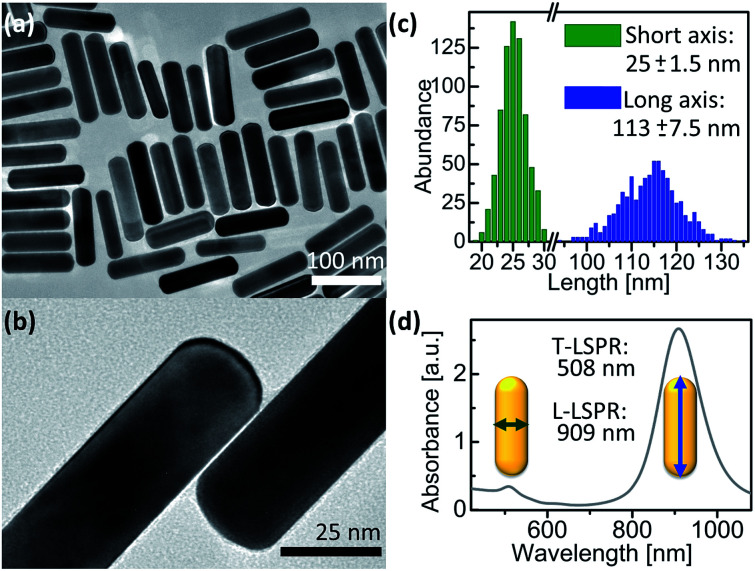
As-synthesized AuNR@CTAB. (a and b) TEM images. (c) Particle size distribution from 726 AuNRs measured by TEM. (d) UV-vis/NIR spectrum.

AuNRs were synthesized using a published protocol.^25^ Transmission electron microscopy (TEM) images of the as-synthesized AuNR@CTAB are shown in Fig. 1a and b. The mean length and width were 115 and 25 nm respectively, both with 6% relative standard deviation (Fig. 1c). As-synthesized AuNR@CTAB exhibited maxima of longitudinal localized surface plasmon resonance (L-LSPR) and transversal resonance (T-LSPR) at 909 and 508 nm, respectively (Fig. 1d).

**Fig. 2:**
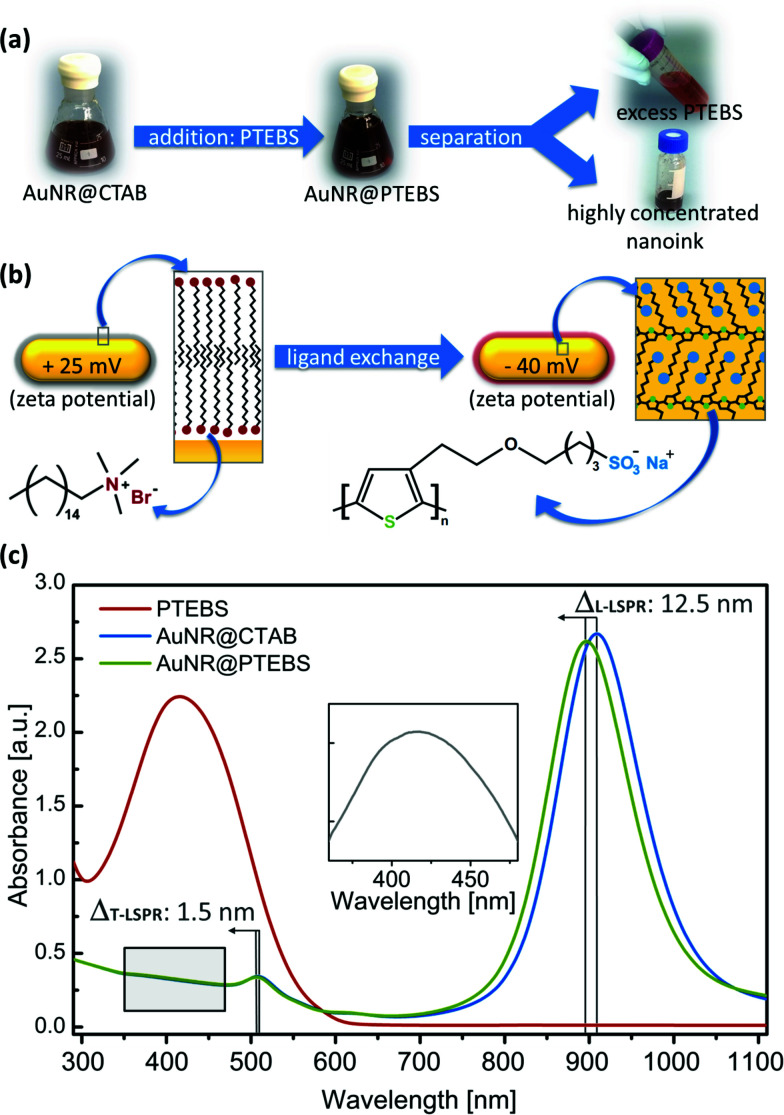
(a) Ligand exchange process: photographs of dispersions before and after ligand exchange. (b) Schematic depiction of the AuNRs surface chemistry before and after ligand exchange. (c) UV-vis/NIR spectra of pure PTEBS, AuNR@CTAB, and AuNR@PTEBS. Inset: difference between the UV-vis spectra of AuNR@PTEBS and AuNR@CTAB.

Fig. 2a schematically depicts the ligand exchange procedure. Washed AuNR@CTAB (excess CTAB below 100 μM)^26^ were incubated with a solution of PTEBS in water. After the ligand exchange, the remaining free (new and old) ligands were separated from the nanorods by centrifugation. The ligand exchange protocol was optimized to provide full coverage of the surface of the studied particles. We determined that a polymer addition to the dispersion equivalent to at least 7 mg m^−2^ (polymer mass/particle surface area) was required to obtain colloidally stable AuNR@PTEBS. We recommend a polymer to surface area ratio equivalent to 10 mg m^−2^ and 8 h incubation for optimal stability. Details on the ligand exchange protocol and its optimization are described in the ESI.†

### Surface chemistry characterization

Fig. 2b illustrates the surface of a nanorod before and after modification. The constitutional formulas of CTAB and PTEBS suggest that the nanorods' surface charge should reverse during a ligand exchange process. The observed change in zeta potential from +25 mV to −40 mV confirms a successful ligand exchange.

The UV-vis spectrum of the AuNR@PTEBS showed a blueshift in both LSPR maxima compared to AuNR@CTAB (Fig. 2c), indicating an increased dielectric constant in the direct vicinity of the nanorods.^27^ We attribute the strong shift to the π-electrons of the conductive polymer that couple with the conduction band of gold. Subtraction of the AuNR@CTAB spectrum from the AuNR@PTEBS spectrum revealed the characteristic absorption band of PTEBS at *λ*_max_ = 415 nm (Fig. 2c, inset) from the polymer attached to the gold surface.

**Fig. 3:**
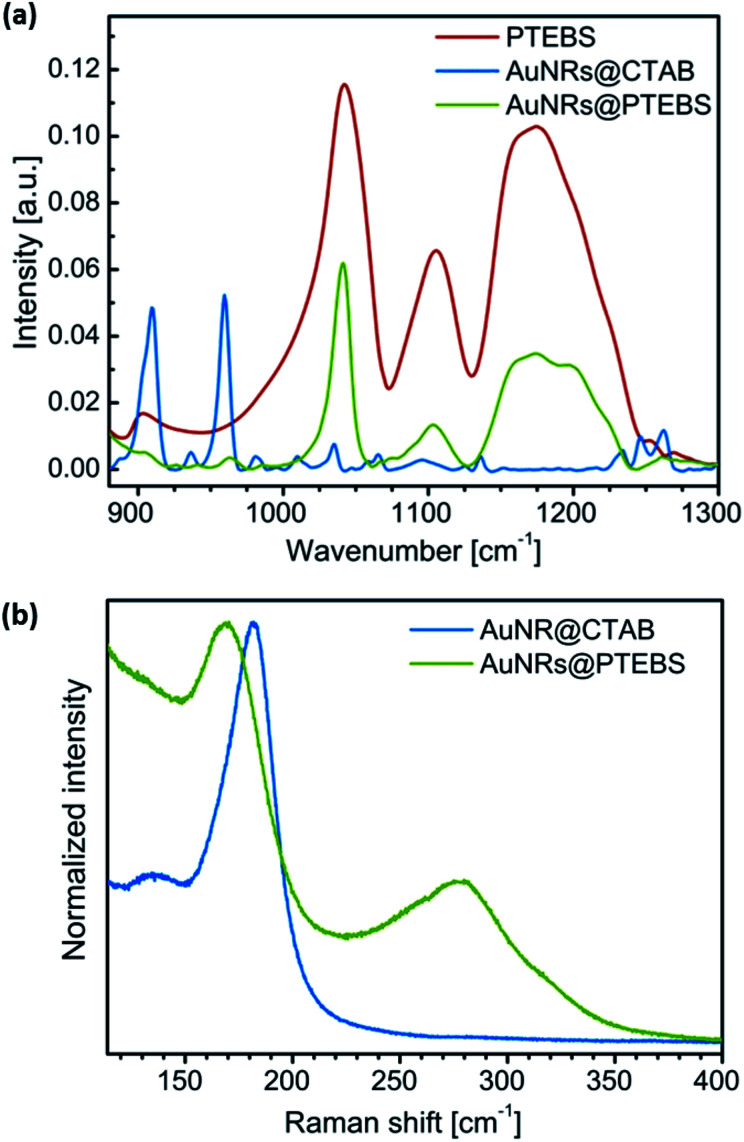
(a) IR spectra of PTEBS, AuNR@CTAB, and AuNR@PTEBS. (b) Raman spectra of AuNR@CTAB and AuNR@PTEBS (*λ*_exc_ = 782 nm) normalized to the maximum intensity.

The completeness of the ligand exchange was confirmed by IR spectroscopy. Fig. 3a compares the fingerprint regions of pure PTEBS, AuNR@CTAB and AuNR@PTEBS. Pure polymer exhibited characteristic vibration bands of the sulfonate group^28^ (*ν*_s_: 1042 cm^−1^; *ν*_a_: 1175 cm^−1^) in the side chain. The original rods, AuNR@CTAB, exhibited two prominent peaks at 910 and 960 cm^−1^. After ligand exchange, AuNR@PTEBS showed only the vibrations of the sulfonate group. The signals in the region of the two prominent peaks from CTAB were negligible, confirming that CTAB has been removed and replaced by PTEBS on the surface of the AuNRs.

Raman spectroscopy was used to determine the PTEBS–Au binding motifs (Fig. 3b). The signal of the Au–bromide bond of AuNR@CTAB occurred at a Raman shift of 182 cm^−1^ as reported in literature.^20,26^ Modified AuNRs did not show this but two other peaks at 172 and 278 cm^−1^. The broad peak at 278 cm^−1^ is in the region where Au–S bonds are typically found.^20^ To clarify whether the peak at 172 cm^−1^ originated from the aromatic ring or from the side chain of PTEBS, AuNR@CTAB were plasma-cleaned until the Au–bromide bond was no longer visible in the Raman spectra. The cleaned surface was dipped into pure thiophene. The resulting spectra (Fig. S4†) evidenced that both peaks found for AuNR@PTEBS arise from thiophene rings adsorbed onto gold. We conclude that PTEBS binds to the AuNRs with its conductive backbone as a multidentate ligand.

**Fig. 4:**
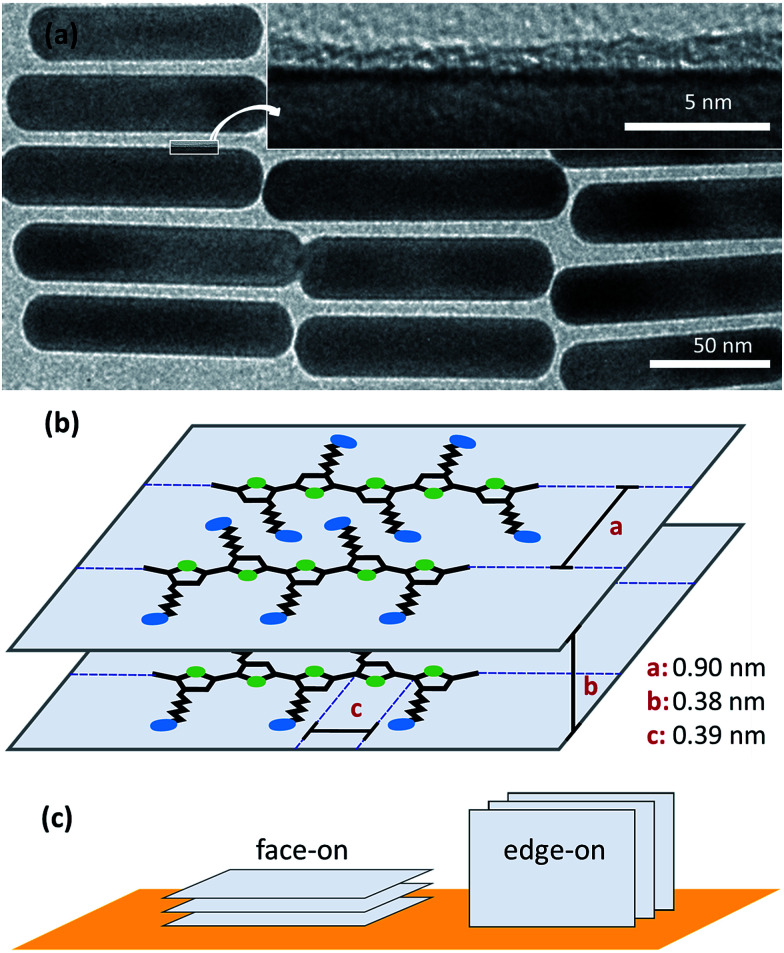
(a) TEM images of AuNR@PTEBS at low and high magnification. (b) Scheme of the polymer chains packing for crystalline PTEBS: *a* is the distance between 2 polymer chains, *b* is the π-stacking distance; *c* is the length of one monomer unit. (c) Two binding types for polymers packed as depicted in (b).

TEM images of AuNR@PTEBS showed dry ligand shells with thicknesses that varied between 0.7 nm and 2.1 nm (Fig. 4a). Thermogravimetric analysis (TGA) on thoroughly washed AuNR@PTEBS resulted in 2.9% mass loss after heating to 800 °C. We converted this value to an average shell thickness using a geometrical model described in the ESI (Table S1†). The packing density of the polymer was estimated for the packing depicted in Fig. 4b with

• the distance of two neighboring polymer chains (*a* = 0.90 nm),^28^

• the π-stacking distance (*b* = 0.38 nm),^29^ and

• the size of one monomer unit (*c* = 0.39 nm).^29^

The dry density of perfectly packed PTEBS on the AuNR surface was 7.5 monomers per nm^3^ (3.5 g cm^−3^) according to this model. This corresponds to a volumetric shrinkage upon ligand removal of 16.1% and an average dry ligand shell thickness of 0.9 nm, in the range of thicknesses observed in TEM.

Conjugated polymers can bind to a surface face-on or edge-on (Fig. 4c).^30^ The binding type affects the electronic properties of the coated particle: the edge-on configuration creates spacing between the conjugated polymer backbone and the metal surface. D. Tanaka *et al.* and Y. Abe *et al.* demonstrated that the spacing between the metal surface and a π-electron system affects electronic coupling.^31,32^ Hence, face-on adsorption of the polymer onto the AuNRs is beneficial for the conductivity of particle–particle interfaces. Our Raman study shows that PTEBS binds face-on with its conductive backbone and not with its side chains. This is in accordance with results previously reported for poly-(3-hexylthiophene) (P3HT), a polymer with the same backbone, that adsorbs face-on on Au (111) surface.^33^ According to the TGA data, each AuNR is surrounded by an average of three polymer layers that bind to the gold and to each other through π-stacking interactions.

We estimated the binding strength of the multidentate ligand from the amount of desorbed polymer measured by inductively coupled plasma mass spectrometry (ICP-MS). A dilute particle dispersion was thoroughly purified to remove free polymer and the closed vessel was shaken at room temperature for one week to reach equilibrium. All particles were then removed by centrifugation. We found 1.1 ± 0.02 ppm of sulphur in the freshly purified, particle-containing sample and 0.4% of it (4.9 ± 0.07 ppb) in the supernatant of the centrifuged sample, demonstrating strong binding of the polymer.

### Colloidal stability

**Fig. 5:**
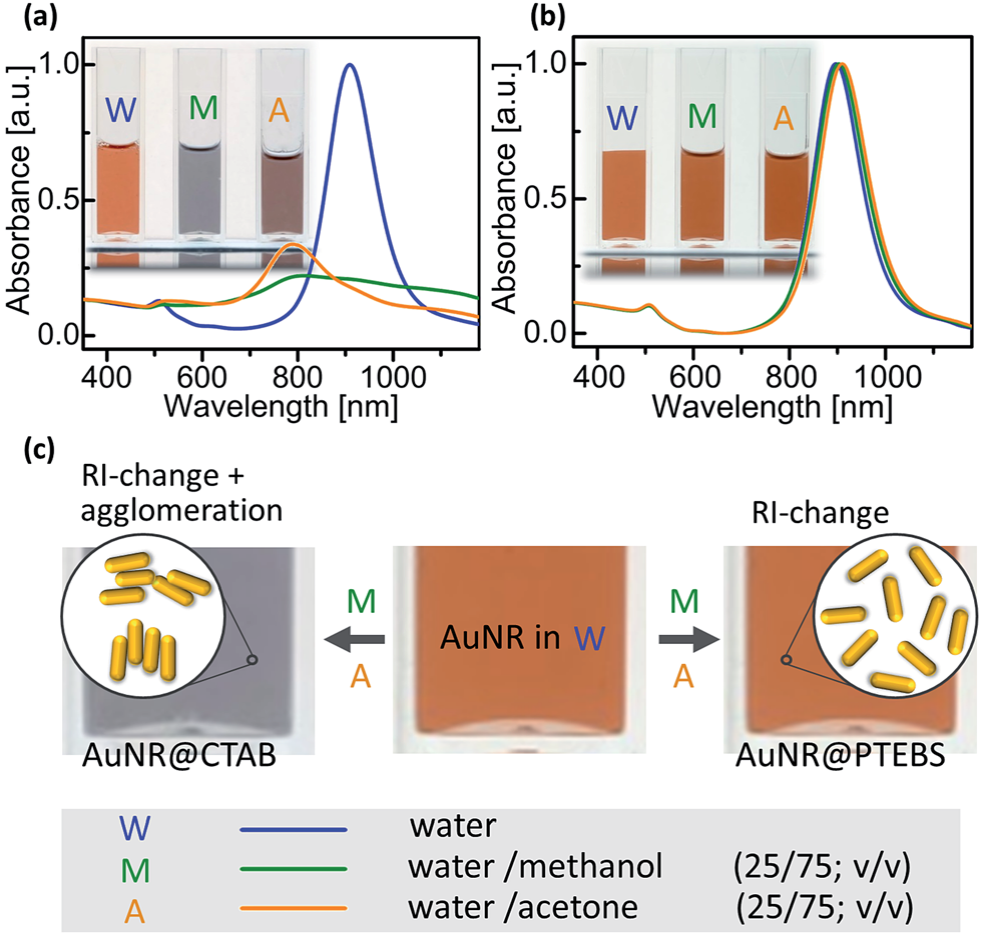
UV-vis/NIR spectra and photographs of dispersions of (a) AuNR@CTAB and (b) AuNR@PTEBS recorded directly after the addition of 75% of water, methanol and acetone, respectively. (c) Schematic depiction of the AuNR's sensitivity towards their assembly and towards the chemical composition of their surrounding media.

Inkjet inks are typically formulated in a mixture of solvents, often water–alcohol mixtures are most convenient.^5^ The poor colloidal stability of AuNR@CTAB in such mixtures limits their use in printed electronics. The rods aggregate even in pure water unless excess CTAB is added, and small amounts of short-chain alcohols or acetone precipitate them.^16^ We compared the colloidal stability of AuNR@CTAB to that of AuNR@PTEBS by centrifuging them, separating the supernatant, and adding different solvents to redisperse them after washing. It was easy to fully redisperse AuNR@PTEBS in short-chain alcohols and in acetone. In a second experiment we introduced rods into solvent mixtures by first dispersing AuNRs in water and adding the 2nd solvent subsequently to a final ratio of 75/25 solvent/water (v/v). Fig. 5a shows that the AuNR@CTAB dispersions responded to the addition of methanol and acetone with a color change that was visible to the naked eye after seconds. The corresponding blueshift and the decrease in intensity in the L-LSPR band (Fig. 5a) are due to side-by-side assembly of the nanorods (Fig. 5c).^34^ Aggregates of AuNR@CTAB in methanol and acetone precipitated irreversibly after minutes. The same experiments with AuNR@PTEBS yielded stable dispersions (Fig. 5b) with a slight red-shift in the L-LSPR bands that is due to the change of refractive index (RI) caused by the second solvent.

We formulated inks from AuNR@PTEBS in water, short chain alcohols, acetone, and their mixtures. Their shelf lifes depended on the polarity of the solvent, as expected for electrosterically stabilized colloids with a zeta potential of −40 mV. Inks in pure acetone or alcohols remained stable for 1–2 weeks. Increasing water content increased stability, and a fully aqueous ink with 100 mg mL^−1^ (12 wt%) particle content was stable under shaking for at least 10 months.

### Conductivity of deposited inks

**Fig. 6:**
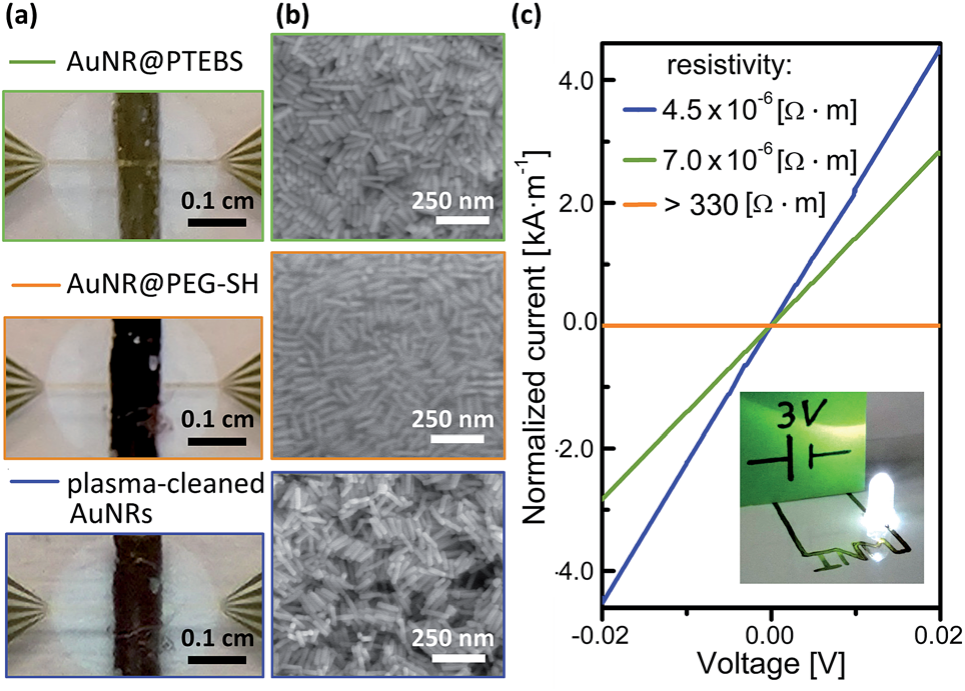
(a) Photographs and (b) SEM images of AuNR lines prepared for electrical testing. (c) *I*–*V* curves with current normalized to film thickness; inset: circuit drawn on glossy paper using a fountain pen loaded with AuNR@PTEBS ink.

The electron transport properties of the modified AuNRs were measured in films deposited from concentrated inks. We compared the results to the properties of AuNRs coated with a non-conductive polymer, *O*-[2-(3-mercaptopropionylamino)ethyl]-*O*′-methylpolyethylene glycol (AuNR@PEG-SH), molecular weight 20 kDa.^20^ Dense lines of AuNR with a thickness of 1 ± 0.2 μm, determined by profilometry, were deposited onto sputtered gold electrodes through masks (Fig. 6a and b) with inks that contained 25 mg mL^−1^ (3 wt%) AuNRs in water/methanol (25/75; v/v). No post-treatment was performed after drying at room temperature (see detailed deposition parameters in the ESI†). Their conductivity was calculated from measured current–voltage (*I*–*V*) curves (Fig. 6c). Note that, in Fig. 6c, current is normalized to the thickness of each line so that the resistivity of the material is equal to the inverse of the slope. AuNR@PTEBS lines were conductive without any further treatment; they exhibited a resistivity of 7.0 × 10^−6^ Ω m, equivalent to a sheet resistance of 276 mΩ sq^−1^ per mil, with a relative standard deviation of 15%. The resistivity of as-deposited lines of AuNR@PEG-SH was above the limits of our measurement (330 Ω m), as expected for inks containing a non-conductive polymer ligand.

A 30 min exposure to a H_2_/Ar-plasma removed the organic ligands from the AuNR@PEG-SH film to below the detection limits of Raman spectroscopy, and the film became conductive. The structure of the nanorods was largely retained during the treatment (Fig. 6b), but the ligand shell removal caused volume shrinkage. The resistivity of the plasma-annealed AuNR line was 4.5 × 10^−6^ Ω m (177 mΩ sq^−1^ per mil). This is about one order of magnitude above the resistivities reported for fully sintered, nanoparticle-based inks where individual particles cannot be distinguished anymore.^35^

The resistivity of the AuNR@PTEBS lines (7.0 × 10^−6^ Ω m) is less than double that of the plasma-treated AuNR. This resistivity is about 250 times that of bulk gold and similar to that of nichrome.^36^ It is considerably lower (about 10 000 times) than that of purely organic conductive polymers and mixtures such as poly(3,4-ethylenedioxythiophene) with polystyrene sulfonate (PEDOT:PSS).^37^

The sinter-free formulation can be applied like a regular ink: we loaded a fountain pen with AuNR@PTEBS at 25 mg mL^−1^ (2.6 wt%) in an isopropanol/water 10/90 (v/v) mixture and drew a circuit on glossy paper (Fig. 6c, inset). The pattern dried within minutes and was conductive enough to power a light-emitting diode (LED).

### Applicability to other polymers

We assessed the versatility of the developed ligand exchange protocol using a structurally different polymer: PEDOT:PSS. This polymer fulfills the requirements for ligands listed above. No changes in the ligand exchange procedure were required to successfully coat AuNRs with PEDOT:PSS.

The resulting AuNR@PEDOT:PSS dispersions possessed a negative zeta potential (−45 mV) similar to that of AuNR@PTEBS and blueshifted LSPRs. They formed stable inks in short-chain alcohols and in acetone. The resistivity of deposited lines of AuNR@PEDOT:PSS was 9.9 × 10^−7^ Ω m (39 mΩ sq^−1^ per mil), 5 times lower than the resistivity of plasma-annealed AuNRs and 7 times lower than the line of AuNR@PTEBS. We believe that the soft PEDOT:PSS shell increases the effective contact area between nanorods. Further experiments have to be performed to clarify the exact mechanism of inter-particle charge transfer.

Long-term stability is a critical property for printed electronics, and PEDOT:PSS is acidic enough to corrode metals.^38^ We performed long-term experiments and stored samples under ambient conditions. Lines of both AuNR@PTEBS and AuNR@PEDOT:PSS retained their electrical performance for at least 1 year. No visible signs of degradation occurred.

### Ink requirements for printing

Printing requires inks with good colloidal stability and suitable rheological properties. Agglomeration leads to inhomogeneous deposition and equipment damage,^5^ inappropriate fluid properties and wetting behavior drastically reduce printing quality.^39^

Our particles are colloidally stable in a wide range of formulations. As an example, we investigated the rheological properties of a formulation that is suitable for inkjet printing, AuNR@PEDOT:PSS, 100 mg mL^−1^ (12 wt%) in isopropanol/water (10/90; v/v) and found a

• density (*ρ*) of 1.2 ± 0.02 mg mL^−1^,

• viscosity (*η*) of 1.46 ± 0.15 cP, and

• surface tension (*γ*) of 54.1 ± 1.1 mN m^−1^.

Commercial piezoelectric printing heads (for example the equipment sold by Microdrop technologies, Germany) often require at least 0.4 cP. The Ohnesorge (Oh) number (eqn (1)) characterizes fluids in inkjet printing:1
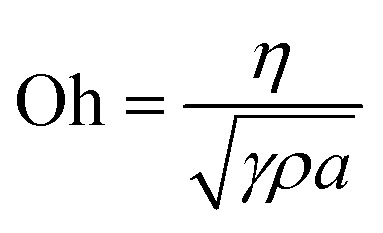
where *a* is a characteristic length, usually the nozzle diameter. Stable printing is possible^39^ for Oh = 0.1–1.0, which implies a upper limit of the nozzle diameter of 3.3 μm for our ink, suitable for very high resolution printing. It is straightforward to tune the viscosity; for example, adding 1 mg mL^−1^ of excess PEDOT:PSS (*i.e.* 0.1 wt% of the liquid ink) increased the viscosity to 4.6 cP, made it suitable for larger low-cost nozzles, and retained the conductivity of the printed lines.

## Conclusions

In summary, thiophene-based conjugated polymers with polar side chains prove to be highly suitable ligands for AuNRs in electronic applications. We developed a simple and straightforward protocol to obtain concentrated, stable colloidal inks suitable for printing. IR and Raman spectroscopy confirmed the complete exchange of CTAB on the AuNRs. PTEBS binds in a face-on configuration with 3 layers of polymer π-stacked on the gold surface in average, a configuration that facilitates electron transport through particle–particle interfaces. Deposited, untreated films reached conductivities comparable to plasma-annealed AuNRs and no signs of degradation were observed after storing them for one year under ambient conditions.

The ligand exchange protocol is also applicable to other polythiophenes as we demonstrated by preparing AuNR@PEDOT:PSS inks. Printed films of AuNR@PEDOT:PSS reached conductivities that surpassed that of AuNR@PTEBS films.

We expect the developed ligand exchange protocol to be applicable to polythiophene derivatives beyond the two examples presented here. The concept is not limited to AuNRs: other conductive or semi-conductive nanoparticles can be coated with conductive polymer ligands to increase inter-particle charge transfer. Nanoparticle-based materials in many applications can profit from this concept. Sintering-free conductive particle packings are a step towards conductive composites of nanoparticles in insulating polymer matrices.

## Supplementary Material

SC-007-C6SC00142D-s001
