# Supplementary material for: Gold nanorods with conjugated polymer ligands: sintering-free conductive inks for printed electronics
Source: Chem Sci. 2016 Mar 15;7(7):4190–6. doi: 10.1039/c6sc00142d (PMC6014069; doi:10.1039/c6sc00142d)
Supplement: SC-007-C6SC00142D-s001 [file SC-007-C6SC00142D-s001.pdf]

Electronic Supplementary Material (ESI) for Chemical Science.
This journal is © The Royal Society of Chemistry 2016

Gold nanorods with conjugated polymer ligands: sintering-free conductive inks for printed electronics

B. Reiser, L. Gonzalez-Garcia*, I. Kanelidis, J. H. M. Maurer and T. Kraus*.

INM-Leibniz Institute for New Materials, Campus D2 2, 66123 Saarbrücken, Germany.

Supporting Information

1. Experimental section

1.1. Materials

All chemicals were used as received without further purification.

Poly[2-(3-thienyl)-ethyloxy-4-butylsulfonate] (PTEBS) (M_w = 40-70 kDa) was purchased from Solaris Chem Inc., (Quebec, Canada).

Poly(3,4-ethylenedioxythiophene) polystyrene sulfonate (PEDOT:PSS) (dry, re-dispersible pellets; resistance: 200-450 Ω/sq) was purchased from Sigma-Aldrich, (Steinheim, Germany).

O-[2-(3-mercaptopropionylamino)ethyl]-O'-methylpolyethylene glycol (PEG-SH) (M_w = 20 kDa) was purchased from Sigma-Aldrich, (Steinheim, Germany).

Thiophene (≥ 99%) was purchased from Sigma-Aldrich, (Steinheim, Germany).

Cetylammoniumbromide (≥ 99%) was purchased from Sigma-Aldrich, (Steinheim, Germany).

Other chemicals were analytical-grade reagents, and all solutions were prepared using Milli-Q water.

Sputtered gold electrodes were purchased from ABTECH Scientific, Inc. (South Carolina 29621, USA).

Silver paste (G3692) was purchased from Plano GmbH (Wetzlar, Germany).

1.2. Characterization

Widths and lengths of the AuNRs were evaluated through transmission electron microscopy (TEM) (JEM 2010, JEOL, Japan) operating at 200 kV. The mean lengths of the short and long axes were calculated by evaluating 726 AuNRs in TEM images. Volume and surface area were calculated using a cylinder with a hemisphere attached at each tip as a model (table S1). Zeta potentials were measured in triplicate on colloidal dispersions containing 1 mM KCl using a Nano ZSP Zetasizer (Malvern, Germany) at 25°C and the data were analyzed based on the Smoluchowski model. Optical characterization of the dispersions was performed with a UV-vis/NIR spectrophotometer (Cary 5000, Varian, CA, USA) in absorbance mode. Fourier transform infrared (FTIR) spectra were recorded in the ATR modus using a Tensor 27 spectrometer (Bruker, Germany). Raman spectra were recorded on a Raman Labram HR Evolution spectrometer (Horiba Jobin Yvon, Germany) using a 782 nm near-IR laser diode. For thermogravimetric analysis (TGA), colloids were washed 3 times with Milli-Q water, dried in an Al2O3 crucible at room temperature, placed in a STA Jupiter 449 F 3 analyzer (Netzsch, Germany), and heated

at 10 °C/min from 50 to 800 °C under an argon atmosphere. Ligand shell volume fractions and thicknesses were calculated from the organic mass fraction obtained by TGA (table S1). Inductively coupled plasma mass spectrometry (ICP-MS) was used to determine the polymer distribution in a AuNR@PTEBS dispersion by comparing the sulfur content before and after removing the nanoparticles by centrifugation. The sulfur content (isotope *m/z:* 32) was measured on an ELEMENT XR mass spectrometer (Thermo Fisher, Germany) with 1250 W plasma power and external calibration with PTEBS standards (R^2: 0.998). Each sample was measured 30 times to calculate the mean value and the standard deviation. Electrical measurements were done with a 2450 Sourcemeter (Keithley Instruments, Ohio, USA); the relative standard deviation was calculated from the averages of 10 measurements on each of 4 samples. Scanning electron microscopy (SEM) images were recorded after film deposition on glass with a Quanta 400 ESEM (FEI, Germany). The thickness of the deposited AuNR patterns was measured with a Surfcom 1500SD3 profilometer (Zeiss, Germany). We removed non-conductive ligands by 30 min exposure to H_2/Ar (5/95 (v/v)) RF plasma (100 W) at 0.3 mbar gas pressure in a PICO plasma reactor (Diener electronic, Ebhausen, Germany). The surface tension of the inks was measured with the pendant drop method in an OCA 35 setup (Dataphysics, Germany) using the Young-Laplace equation. A digital camera was used to record the shape of the pendant drop. Mean values and the standard deviations were calculated from a minimum of 200 measured surface tension values. Dynamic viscosity of the ink at room temperature was measured in the rotation mode of a PHYSICA MCR 300 modular compact rheometer (Anton Paar, Germany). Mean values and the standard deviations were calculated from a minimum of 10 shear experiments.

Table S1. Properties of AuNR@PTEBS.

**Au core**			**Ligand shell**		
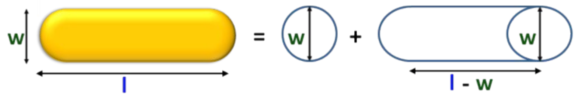			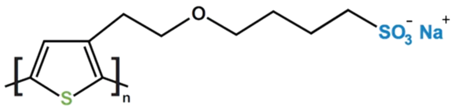		
**Length (TEM)**	113	nm	**Mass fraction (TGA)**	2.9	%
**Width (TEM)**	25	nm	**Volume fraction (calculated)**	16.1	%
**Density (literature [1])**	19.3	g/cm^3	**Density (literature [2,3])**	3.5	g/cm^3
**Volume (calculated)**	5138	nm^3	**Volume (calculated)**	8300	nm^3
**Surface area (calculated)**	8875	nm^2	**Thickness (calculated)**	0.9	nm

1.3. Nanoparticle synthesis and ligand exchange

Gold nanorods (AuNR@CTAB) were prepared following a published protocol[4] and washed to ensure an excess CTAB concentration of below 80 µM (Note that literature[5] recommended concentrations below 100 µM, however we found that concentrations below 80 µM were preferable in our case). The washed AuNRs were immediately incubated with the new ligand at room temperature under vigorous stirring in order to prevent agglomeration due to the low CTAB concentration. After the incubation time (2-8 h), the AuNR@PTEBS were separated from the excess PTEBS by centrifugation. PTEBS remaining in the supernatant was quantified by UV-vis spectroscopy to estimate the amount of polymer adsorbed on the nanorods (figure S2). The remaining concentration was used to calculate the PTEBS surface coverage of the AuNRs and thus, the polymer/surface ratio required to form a sufficiently dense polymer ligand layer. We found that AuNRs could be redispersed only for PTEBS ratios above 5.6 mg/m^2. Ratios below 6.7 mg/m^2 provided AuNRs that were stable but only for a few hours to a few days. Larger ratios yielded AuNRs that were stable for weeks. Figure S1 compares colloidal dispersions after ligand exchange with insufficient (left) and sufficient (right) PTEBS. We attribute the differences in colloidal stability to the ligand surface density. Table 2 shows PTEBS surface coverage calculated from the amount of PTEBS remaining in the supernatant. The values indicate an increase in coverage for up to a PTEBS/surface ratio of 9.0 mg/m^2, with saturation for greater values.

We also studied the incubation time required for ligand exchange using the blueshift in the AuNRs L-LSPR band. No change in the L-LSPR band occurred after 8 h regardless of the PTEBS excess employed. In summary, we recommend a reaction time of 8 h and a PTEBS/surface ratio equivalent to 9-10 mg/m² to obtain complete ligand exchange reliably. For example, to obtain 10 mg/mL of AuNR@PTEBS, 5 mL of AuNR@CTAB in a concentration of 20 mg/mL (equivalent surface area of 0.886 m^2) was incubated with 5 mL of a 1.6 mg/mL of PTEBS solution.

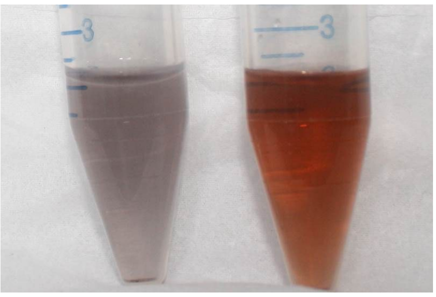

Figure S1. Photograph of dispersions after redispersion in Milli-Q water subsequently to ligand exchange with a PTEBS/Au surface area ratio of left: 2.8 mg/m² and right: 11.3 mg/m².

1.4. Calibration of PTEBS concentration measurements

Aqueous solutions containing between 0.1 and 250 µg/mL of PTEBS were prepared and their integrated absorbance between 310 and 550 nm was measured. The peak area showed a linear dependence of the PTEBS concentration throughout the full concentration range (figure S2). We used the PTEBS concentration decrease in the supernatant and the known amount of gold surface area in the reaction mixture to calculate PTEBS surface coverage.

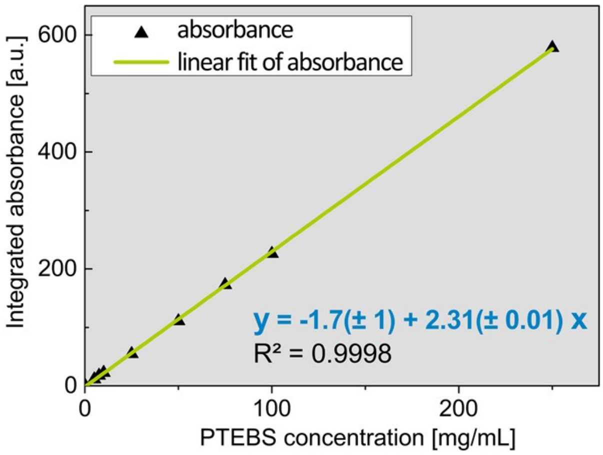

Figure S2. Calibration curve for aqueous solution of PTEBS: Integrated absorbance from 310 and 550 nm versus PTEBS concentration.

Table S2. Quantification of PTEBS adsorbed onto AuNRs using the external calibration curve (figure S2). Incubation time: 2 h.

PTEBS concentration [μg/mL]	PTEBS mass –Au surface ratio [mg/m²]	PTEBS in the supernatant [μg/mL]	PTEBS adsorbed onto AuNRs [μg/mL]	Estimated AuNR surface coverage [mg/m²]
**250**	**2.8**	**193.5**	**56.5**	**0.6**
**500**	**5.6**	**391.7**	**108.3**	**1.1**
**600**	**6.7**	**481.1**	**118.9**	**1.2**
**800**	**9.0**	**632.7**	**167.3**	**1.7**
**1000**	**11.3**	**833.6**	**166.4**	**1.7**

1.5. Layer preparation

AuNR patterns were prepared by depositing 10 μL of the inks (25 mg/mL, 3 wt% gold content, dispersed in a mixture of methanol/water (60/40; v/v)) on a glass substrate. PDMS stencil masks, placed on the substrate and separated 1 mm, were used to define the deposition area. Lines were dried at room temperature overnight to ensure complete drying before performing the electrical tests.

The cartridge of a commercial fountain pen was loaded with sintering-free ink containing 25 mg/mL (3 wt%) of gold dispersed in a mixture of isopropanol/water (10/90; v/v). Glossy paper (for photographic prints) was used as the substrate. A light-emitting diode (LED) and batteries were connected to the circuit with silver paste. Photographs of the writing and the circuit are shown in Figure S3.

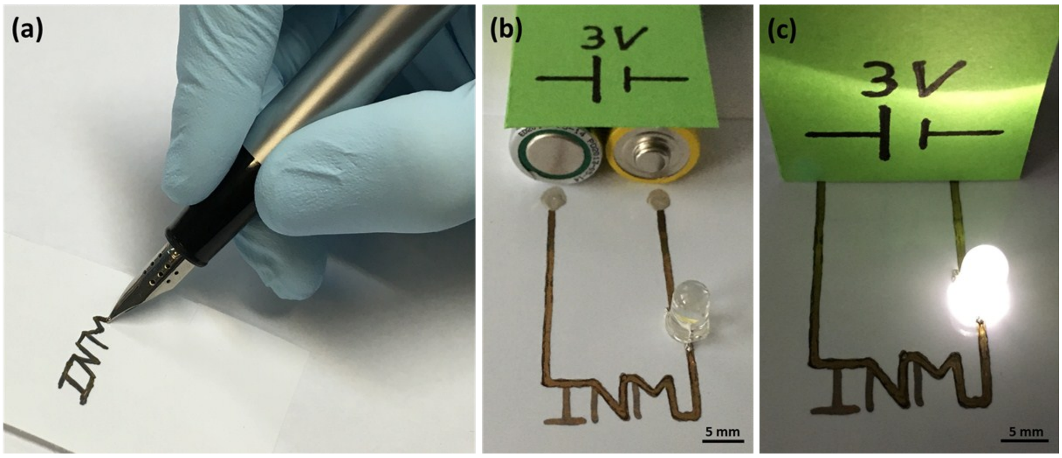

Figure S3. Photographs of (a) writing, (b) the open circuit, and (c) the closed circuit.

2. Comparison of PTEBS and thiophene adsorbed onto AuNRs

We deposited AuNR@CTAB films, subjected them to H_2/Ar plasma for 30 min, and dipped them into pure thiophene for 5 minutes to create AuNR@Thiophene films. Residual thiophene was evaporated at room temperature before the measurement.

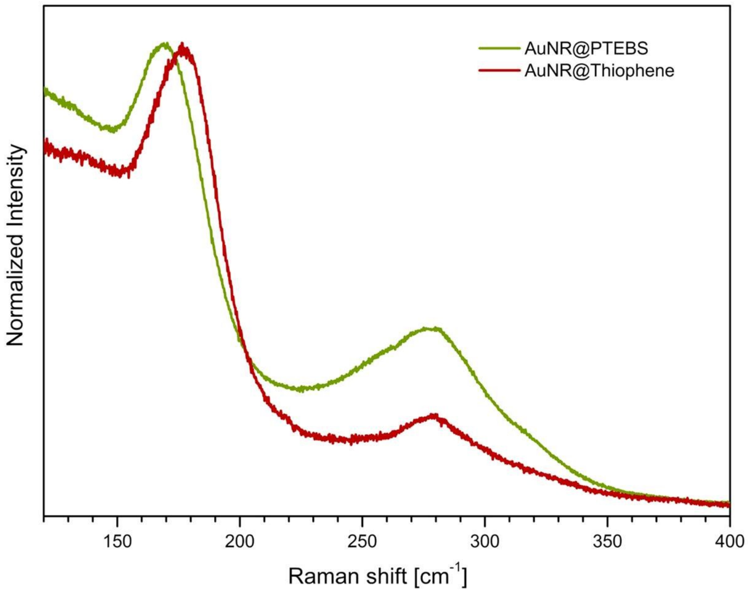

Figure S4. Normalized Raman spectra of AuNR@PTEBS and AuNR@Thiophene.

3. Literature

1 D. R. Lide, Ed., *CRC Handbook of Chemistry and Physics*, CRC Press. Boca Raton, Florida, 84th edn., 2003.

2 R. Colle, G. Grosso, A. Ronzani and C. M. Zicovich-Wilson, *Phys. status solidi*, 2011, **248**, 1360–1368.

3 S. Zhang, C. I. Pelligra, G. Keskar, J. Jiang, P. W. Majewski, A. D. Taylor, S. Ismail-Beigi, L. D. Pfefferle and C. O. Osuji, *Adv. Mater.*, 2012, **24**, 82–87.

4 X. Ye, C. Zheng, J. Chen, Y. Gao and C. B. Murray, *Nano Lett.*, 2013, **13**, 765–771.

5 M. Tebbe, C. Kuttner, M. Männel, A. Fery and M. Chanana, *ACS Appl. Mater. Interfaces*, 2015, **7**, 5984–5991.
